# Research on the prediction of longevity from both individual and family perspectives

**DOI:** 10.1371/journal.pone.0263992

**Published:** 2022-02-18

**Authors:** Lvqing Miao, Suyu Yang, Yuye Yi, Peipei Tian, Lichun He

**Affiliations:** 1 Department of Psychology, Institute of Special Environmental Medicine, Nantong University, Nantong, Jiangsu Province, China; 2 School of Psychology, Shandong Normal University, Jinan, Shandong Province, China; 3 School of Education Science, Nantong University, Nantong, Jiangsu Province, China; University of Palermo, ITALY

## Abstract

Increasing human longevity is of global interest. The present study explored the prediction of longevity from both individual perspective and family perspective based on demographic and psychosocial factors. A total of 186 longevous family members and 237 ordinary elderly family members participated in a cross-sectional study, and a sample of 62 longevous elderly and 57 ordinary elderly were selected for comparative research. The results showed that it was three times more female than male in longevous elderly group. Up to 71.2% of longevous elderly had no experience in education, which was significantly lower than that of ordinary elderly. Due to such extreme age, more widowed (81.4%) elderly than those in married (18.6%). Less than one-seventh of the longevous elderly maintained the habit of smoking, and about one-third of them liked drinking, both were significantly lower than that of ordinary elderly. In terms of psychosocial factors, longevous elderly showed lower neuroticism and social support, while higher extraversion, compared with the ordinary elderly. However, there were no significant differences between the two family groups in demographic and psychosocial variables, except longevous families showing lower scores in neuroticism. Regression analysis found that neuroticism, social support and smoking habit had significant impact on individuals’ life span, then, neuroticism and psychoticism were the key factor to predict families’ longevity. We conclude that good emotional management, benign interpersonal support, and moderation of habits are important factors for individual longevity, and the intergenerational influence of personality is closely related to family longevity.

## 1. Introduction

With the decrease of the population fertility rate and the increase of life expectancy, many countries have gradually entered a state of population aging, by the end of 2019, the proportion of the world’s population in the elderly has reached 9%, and China is the second most aging Asian country outside of Japan. The population’s degree of aging ranks tenth in the world, and the total number of aging individuals ranks first. According to the seventh national census data from China’s National Bureau of Statistics, the proportion of the population aged 60 and over reached 18.7%, of which the proportion of individuals over 65 years old reached 13.5%. With continued rapidly aging population, the elderly have become a group that cannot be ignored, and the demand for aging care has risen considerably, China is currently facing an unprecedented complex health demand [1].

The ‘elderly health promotion action’, which is part of the strategy of ‘healthy China action (2019–2030)’ formulated by the National Health Commission, is worthy of our attention, and with the transformation of modern medical models, the concept of health is no longer limited to physical health but also includes mental health and social functional integrity. Elderly individuals’ mental health is not only an important psychological research topic but also a pressing social issue. There are unique standards of mental health for the elderly population. A research report on mental health of elderly emphasizes the mental health standards of the elderly from five aspects: normal cognitive functioning, positive and stable emotions, appropriate self-evaluation, harmonious interpersonal relationships, and good environmental adaptation [2]. From these standards, we can easily find that psychosocial factors are key influences on elderly people’s mental health.

It is commonly known that mental health is closely related to age [3]. Increasing human health and longevity is of global interest [4]. Human longevity and health are affected by many factors, including gender and genetics [5], natural environment [6–8], social support [9], religiousness [10], sleep problems [11] and some individual traits [12–16]. Among these factors, heredity, individual personality and social support are extremely influential.

Longevity is significantly influenced by genetic characteristics and familial aggregation [17]. Some studies have demonstrated that the influence of heredity on lifespan is small before the age of 60 then increases gradually [18], especially after the age of 100 [17]. The offspring of centenarians hold lower susceptibility to a broad range of age-related diseases [19, 20] and tend to live longer than their peers [21]. Siblings of centenarians might also live past age 100 [22]. Centenarians tend to be less afflicted with age-related, terminal diseases [23]. For example, most longevous people do not have cardiovascular disease, diabetes, cancer and so on, which may be determined majorly by heredity [24].

Personality is also recognized as an important variable associated with longevity and with elderly people’s mental health. Terracciano and colleagues [25] conducted a nearly five-decade longitudinal study with a large sample of generally healthy individuals and found that longevity was associated with being conscientious, emotionally stable and active; conversely, researchers have found that low conscientiousness and low emotional stability are related to reduced healthy life expectancy [26, 27]. Extraversion has predicted old-age social competence for both men and women, and conscientiousness has predicted men’s old-age productivity [28]. Studies also have demonstrated that longevous elderly, especially centenarians, tend to have low levels of neuroticism and high levels of extraversion, conscientiousness, competence and trust [28, 29].

Additionally, studies have indicated that family emotional support has significant effects on both physical and mental health [30, 31]. One investigation found that co-residence with adult children is beneficial for the psychological well-being of the ‘oldest-old’—that is, those aged 80 and older [32]. Despite physical health deterioration, the oldest-old often report good mental health and have positive perceptions of their health status [33–35]. This might be made possible by the psychosocial resources that promote well-being during the process of aging. Among these resources, social support can moderate the impact of stressful circumstances and plays an important role in the mental health and personality development of the elderly [36]. Both objective (living arrangements and received support) and subjective (perceived support) forms of social support have an impact on elderly people’s survival and health [37].

Centenarians are considered as the paradigm for successful aging, as they have largely delayed or escaped major illnesses [38]. As a weak group being divorced from the mainstream production field, longevous elderly people often reflect more conceptual conflicts and pressures, and their mental health requires more attention. The age threshold used to define longevity varies by study but is typically≥85 years old [5],exceptional longevity may be considered age ≥95 years old [39], and with reference to the research of Chinese scholars [40, 41], we defined longevous elderly as those age ≥90 (longevous elderly group). Then, we designated the age range of ordinary elderly group as 60–89 years old (ordinary elderly group). Simultaneously, we set the criteria for a longevous family as follows: a family with at least one person age ≥90 and two or more immediate relatives (e.g., sons, daughters, grandsons or granddaughters) living together. The standard ordinary elderly families were three or more family members living near the longevous families, and none of their members has lived more than 90 years in the past five generations.

Despite studies [42] on the factors affecting elderly people’s mental health and longevity, it is lack of a systematic research on the prediction of longevity from family perspective. We compared longevous elderly and ordinary elderly individuals on demographic and psychosocial variables (personality and social support), and explored the prediction of longevity from both individual perspective and family perspective. We posited three hypotheses:

1. There will be significant differences in demographic and psychosocial factors between longevous and ordinary elderly individuals.
2. The demographic differences between the longevous families and ordinary elderly families may not be significant, but obvious differences on personality characteristics.
3. There would be different models for predicting longevity from individual and family perspective, but personality would be the most strong predictors in both models.

We did in order to provide suggestions from a psychosocial perspective for people to prolong and improve the quality of their lives. At the same time, it was also hoped that the government and society could pay more attention to the mental health of long-lived groups, and gradually realized the transformation from disease-based treatment model to the elderly-centered comprehensive care model.

## 2. Methods

### 2.1 Samples

G-Power 3.1.9.2. Power analysis was used to determine the sample size of the present study, which indicated that a total of 114 participants (with one group of 57 samples) was needed to detect a medium effect size (Cohen’s d = 0.53) when α = 0.05 for a power of 0.8 with 1 group, using a t-test (two independent samples). Therefore, we recruited participants from 62 longevous families and 88 ordinary elderly families in Nantong, China. Among them, 62 longevous elderly (M = 98.74, SD = 5.24) and 57 ordinary elderly (M = 73.47, SD = 8.37) participated in the individual survey; additionally, we collected family data of 186 longevous family members (M = 50.49, SD = 17.74) and 246 ordinary elderly family members (M = 50.46, SD = 15.33) for comparative research purposes.

We applied the following exclusion criteria: (a) history of self- or other-reported cognitive impairment; (b) currently suffering from cognitive disorders; and (c) self- or other-reported total deafness. No participants were excluded for these reasons.

We secured written consent from all participants and obtained approval for the research from the Nantong University Academic Ethics Committee.

### 2.2 Procedure

Field investigation and face-to-face interview techniques were applied to assess the psychosocial characteristics of longevous elderly and their families. We utilized a combination of cluster sampling and random sampling to select suitable target participants. Specifically, we first selected the longevity communities, and then randomly selected a family with a longevous elderly over 90 years old, a longevous family sample was composed of a longevous elderly and more than two immediate relatives living together. In order to control the differences in life experience or living habits, the sampling standard of the ordinary elderly family (ordinary elderly family) was: living around longevous families, that was, neighbors of longevity elderly, and there wasn’t a longevous elderly in five generations. Four research assistants administered this survey. To obviate age and cultural limitations, the assistants explained in detail the various test indicators and the response formats of the scales. Then, they used local dialect to read the items one-by-one and collected the longevous elderly’s answers. Other participants with the ability to complete the scales were required to complete them independently.

### 2.3 Measurement

#### 2.3.1 Demographics

Demographic items included gender (male/female), age (all >25), education level (high school and beyond, basic education (elementary and junior high school) and no schooling), marital status (married, widowed, divorced, still single and others), self-reported health status (good, generally good, poor; and for the older adults age over 70, due to their lack of education about the health status without proper medical accessibility, the survey question was how they felt about their health), habits (smoking, drinking) and household income (monthly income of each person in a family).

#### 2.3.2 Eysenck Personality Questionnaire-Revised, Short Scale or Chinese (EPQ-RSC)

The EPQ-RSC was revised since 1997 on the basis of Eysenck Personality Questionnaire-Revised, Short Scale (EPQ-RS) [43]. It included four subscales of 48 total items. The scale had proved to have good reliability and validity, the test-retest reliability was 0.67–0.88, and the internal consistency coefficient was 0.58–0.78. All the items were scored 0–1, 1 for "yes" and 0 for "No", then we converted the score for each subscale into a standard T-score (T = 50+10X, The original score of the tested individual–the average score of his/her group / the standard deviation of the score of the group) [44].

The subscales are summarized below:

1. Extraversion (E): It contained introverted and extraverted dimensions, reflecting the tendencies to be sociable or to be alone, respectively. Individuals with high scores tend to be energetic, optimistic, quick-reacting, adventurous, less practical and impulsive. Individuals with low scores tend to be quiet, conservative, reliable and pessimistic.
2. Neuroticism (N): ThIt was also known as emotional stability. It reflected normal, not pathological, behaviour. Individuals with high scores tend to be emotionally unstable, anxious, irritable and often depressed. Individuals with low scores are emotionally stable, react slowly or weakly and are not prone to anger.
3. Psychoticism (P): Also called stubbornness and pragmatism, but didn’t allude to mental illness. High scores indicate loneliness, unkindness or indifference toward others, difficulty adapting to the external environment and enjoyment of novel activities. Low scores denote the opposites of these tendencies.

#### 2.3.3 Social Support Rating Scale

Xiao created the Social Support Rating Scale in 1994 [45]. The scale divided social support into three dimensions: subjective support, objective support and utilization of social support, and comprised 10 total items. The consistency coefficient of the total score and each item over a two-month period was 0.89–0.94 (p < 0.01). The subscales were described below: (1) Subjective support refers to the emotional experience and satisfaction wherein an individual feels respected, supported and understood in society. (2) Objective support refers to visible or actual support, including direct material assistance and the existence of and participation in social networks. This type of support is independent of the individual’s subjective perceptions of support. (3) Utilization of support denotes the extent to which individuals use their resources.

### 2.4 Data analysis

We analyzed the data in several steps. First, we examined descriptive statistics of the demographic variables for individual and family subsamples. Second, we utilized t-tests to examine age differences in the subsamples and chi-square tests to examine differences in the other demographic variables. Third, t-tests were used to examine the differences in personality and social support between longevous elderly and ordinary elderly, as well as longevous and ordinary elderly families. Finally, we used logistic regressions to evaluate the predictors of longevity from both individual perspective and family perspective.

## 3. Results

### 3.1 Individual differences on demographic and psychosocial variables

As shown in Table 1, we displayed the demographics for both longevous elderly group and ordinary elderly group. Among the longevous elderly, only 22.6% were male, and percentage of female was three times more than male. Up to 71.2% of longevous elderly had no experience in education, which was significantly higher than the proportion of educated elderly. Longevous elderly differed significantly in terms of marital status, more widowed (81.4%) elderly than those in married (18.6%), which was largely related to their age. Most of them self-reported that they were in good health, only 3.3% were in poor health. Less than one-seventh of the longevous elderly maintained the habit of smoking, and about one-third of them liked drinking. Three-quarters of longevous elderly believed that their household income was low.

**Table 1:** Demographics for elderly subsamples (%).

Demographic Variable		Longevous Elderly	Ordinary Elderly
Gender	Male	22.6	52.6
	Female	77.4	47.4
Age	Mean (*SD*)	98.74(5.24)	73.47(8.37)
Education	High school and beyond	3.4	25.9
	Basic education	25.4	60.3
	No schooling	71.2	13.8
Marital Status	Married	18.6	69.1
	Widowed	81.4	29.1
	Divorced	0	0
	Single and others	0	1.8
Health Status	Good	82.0	75.5
	Generally good	14.8	22.6
	Poor	3.3	1.9
Habits	Smoking	13.1	41.1
	Drinking	29.5	50
Household Income	≤ RMB 2000	75.6	85.4
	≥ RMB 2000	24.4	14.6

Note: Statistical indicators were valid percentages (Longevous elderly information was unreported for marital status of 3, the health status of 1, the habits of 2, the household income of 7; ordinary elderly information was unreported for marital status of 2, the health status of 4, the habits of 2, the household income of 9); Chi-square tests were used to compare the differences of the rate; Age differences in continuous variables were tested using t-test.

However, compared with ordinary elderly, there were significant differences between the two groups. After controlling the sex ratio of the ordinary elderly group (since it could minimize the error caused by the imbalance of the sex ratio), the proportions of longevous elderly receiving education were significantly lower than that of ordinary elderly (*χ*^*2*^_High school and beyond_ = 18.24, *p* < 0.001; *χ*^*2*^_Basic education_ = 14.41, *p* < 0.001) and the proportion without any educational experience was significantly high (χ^2^ = 38.22, *p* < 0.001). Because of the difference of age (*t* = 19.59, *p* < 0.001, Cohen’s d = 0.875), the percentage of married longevous elderly was significantly low (*χ*^*2*^ = 28.41, *p* < 0.001), and that of widowed was significantly higher (*χ*^*2*^ = 24.58, *p* < 0.001). Besides, the proportions of bad habits in the two groups were significantly different (*χ*^*2*^_Smoking_ = 14.52, *p* < 0.001; *χ*^*2*^_Drinking_ = 5.00, *p* < 0.05).

Then, a series of independent sample *t*-tests revealed significant group differences on psychosocial variables in Table 2. Longevous elderly showed higher extraversion (*p* < 0.05) but lower neuroticism (*p* < 0.01) in personality characteristics. While for social support, there were significant differences in scores of subjective support (*p* < 0.05) and total support (*p* < 0.05), with longevous elderly scoring lower on these variables than ordinary elderly.

**Table 2:** Individual differences on psychosocial variables.

Psychosocial Variable	*M* (*SD*)		*t*	Cohen’s d
	Longevous Elderly	Ordinary Elderly		
Extraversion	53.10 (9.79)	49.39 (9.15)	2.11*	0.390
Neuroticism	40.94 (7.29)	45.55 (9.56)	-2.96**	0.543
Psychoticism	49.74 (10.29)	48.46 (10.94)	0.66	-
Objective Support	9.19 (4.46)	10.24 (4.23)	-1.32	-
Subjective Support	18.23 (8.30)	21.74 (7.21)	-2.45*	0.452
Support Utilization	7.23 (3.25)	8.02 (2.48)	-1.48	-
Total Support	34.65 (3.77)	40.00 (11.82)	-2.27*	0.417

Note:

* *p* < 0.05

** *p* < 0.01.

### 3.2 Family differences on demographic and psychosocial variables

As for family differences, we focused on the differences between the two groups on demographic and psychosocial variables (Table 3). We had relatively balanced the gender and age range of the two groups (*p* = 0.987). As shown in Table 3, the distributions of marital status and household income level across two groups were similar. The rate of longevous families receiving education of high school and beyond is slightly lower. The percentage of smoking and drinking in longevous families was slightly lower than that of ordinary elderly families. Accordingly, compared with ordinary elderly families, the percentage of self-reported good health was higher among longevous families. However, chi-square tests determined that there were no significant differences between longevous and ordinary elderly families in education, marital status, health status, habits and household income.

**Table 3:** Demographics for family subsamples (%).

Demographic Variable		Longevous Families	Ordinary elderly Families
Gender	Male	58.1	55.1
	Female	41.9	44.9
Age	Mean (*SD*)	50.49 (17.74)	50.46 (15.33)
Education	High school and beyond	50.3	62.8
	Basic education	43.3	33.8
	No schooling	6.4	3.4
Marital Status	Married	81.0	86.1
	Widowed	4.3	7.1
	Divorced	0	1.3
	Single and others	14.7	5.5
Health Status	Good	90.6	82.8
	Generally good	8.3	15.5
	Poor	1.1	1.7
Habits	Smoking	28.5	34.0
	Drinking	34.9	41.7
Household Income	≤ RMB 2000	63.7	61.7
	≥ RMB 2000	36.3	38.3

Note: Statistical indicators were valid percentages (Longevous families information was unreported for marital status of 2, the health status of 6, the household income of 18; ordinary elderly families information was unreported for marital status of 9, the health status of 14, the habits of 11, the household income of 24); Chi-square tests were used to compare the differences of the rate, age differences in continuous variables were tested using t-test.

Table 4 displayed the results with respect to family differences on the psychosocial variables. Longevous families’ neuroticism scores were significantly lower than those of ordinary elderly families (*p*< 0.05, Cohen’s d = 0.286), but there were no significant differences between the two types of families on other personality characteristics and social support.

**Table 4:** Family differences on psychosocial variables.

Psychosocial Variable	*M* (*SD*)		*t*
	Longevous Families	Ordinary elderly Families	
Extraversion	51.49 (9.76)	51.85 (9.74)	-0.38
Neuroticism	43.44 (9.21)	46.11 (9.48)	-2.93**
Psychoticism	49.81 (10.38)	48.44 (9.76)	1.40
Objective Support	10.75 (3.94)	10.92 (3.89)	-0.44
Subjective Support	23.76 (6.80)	23.81(6.16)	-0.08
Support Utilization	8.09(2.33)	7.94 (2.57)	0.58
Total Support	42.61 (9.63)	42.68(9.95)	-0.08

Note:

** *p* < .01.

### 3.3 Variables associated with longevity

We used binomial logistic regression to determine the predictive validity of certain indicators and establish a comprehensive evaluation model with respect to longevity (Table 5). In terms of individual model, longevous elderly was assigned a value of 1 and ordinary elderly of 0, then we used the forward LR variable entry method, the standard was that the probabilities of entry and deletion was 0.05 and 0.1 respectively. The regression analysis revealed that neuroticism, total social support and smoking habits all significantly impacted individual lifespan. As the score of neuroticism and total support decreased, the possibility of individual longevity increased, and compared with non-smokers, smokers were less likely to live a long life. The results also showed that the overall prediction accuracy of the model for individual longevity was 71.8%.

**Table 5:** Prediction model of longevity.

Prediction Model	Variable	OR	95% CI	*p*
Individual Model	Neuroticism	0.92	(0.87, 0.97)	0.001
	Total Support	0.96	(0.92, 0.99)	0.017
	Smoking	0.17	(0.06, 0.45)	0.000
Family Model	Neuroticism	0.96	(0.94, 0.99)	0.001
	Psychoticism	1.02	(1.00, 1.04)	0.040

Similarly, as for family model, we used the same method and it turned out that neuroticism and psychoticism significantly influenced family longevity. As the score of neuroticism decreased, the possibility of family longevity increased, while high psychoticism could predict family longevity to a certain extent. The overall prediction accuracy of the model for family longevity was 60.8%.

## 4. Discussion

The present study focused on the perspectives from individual and family to investigate the predictors of longevity. The results showed that no significant difference in demographic characteristics among families, but individuals did differ in terms of gender, marital status and habits (smoking and drinking). More than 75% of longevous elderly were women, and more than 80% were widowed, which was strongly correlated with age [46]. Taylor et al. [47] studied the benefits of smoking cessation for longevity and determined the life extension obtained from stopping smoking at various ages. They and other researchers [48] have concluded that stopping smoking as early as possible was important, but cessation at any age provides considerable life extension. On the relationship between drinking and longevity, opinions have not been uniform. Some have argued that moderate wine drinking increases longevity, reduces the risk of cardiovascular diseases and does not appreciably influence the overall risk of cancer [49, 50]; in contrast, some have posited that this is not necessarily because of the adjustment to some biochemical substances in the body [51, 52].

The personality traits examined in this study included extraversion, neuroticism, and psychoticism. Several studies have established the relationship between personality and longevity [13, 25, 27, 28, 43, 53]. Additionally, previous studies have found that neuroticism was closely related to elderly people’s mental health [26, 27, 54, 55], and personality especially neuroticism influenced aging attitudes, which in turn affect well-being [56]. Our findings that compared with ordinary elderly, longevous elderly behave more outgoing, and longevous individuals’ and families’ neuroticism was lower than that of their ordinary counterparts. Consistent with previous studies, our research verified that members of the longevous families, including the longevous elderly individuals themselves, tend to exhibit more stable and positive emotions and behaviors. Participants with low neuroticism scores tend to exhibit a milder character and more altruistic behaviors, control their desires and have fewer mental illnesses.

Social support refers to the material and spiritual help that individuals can obtain from those in their social networks, defined in terms of family living arrangements, care giving and social interaction [57]. Studies have demonstrated that improving social support for the elderly can reduce their loneliness [58], improve quality of life [59], subjective well-being [60] and life satisfaction [61], and help to extend their lives [62]. However, our study found that, compared with ordinary elderly, subjective and overall support for longevous elderly were significantly lower. This result might be explained by several factors. First, longevous elderly in our study are mostly women living alone, whose social supports come primarily from their spouses [63], and older female have higher self-esteem than male [64], even if life is unsatisfactory, they are unwilling to accept support from others therefore, widowhood [32] and high self-esteem may the main factors that reduce social support, because the elderly are in need of self-esteem. Second, compared to receiving social support, some researchers believe that providing social support may be more beneficial. They found that mortality was significantly reduced for individuals who reported providing instrumental support to friends, relatives and neighbors, as well as for individuals who reported providing emotional support to their spouse; receiving support had no effect on mortality once giving support was taken into consideration [65], which suggesting that further research can pay attention to the relationship between good deeds and longevity. To further explore the predictive factors of longevity, we examined demographics, personality traits and social support, then we constructed evaluation models of longevity from both individual and family perspectives. We found that neuroticism, social support and smoking habits significantly predicted individual longevity. In other words, elderly people who had more stable emotion state, less smoking habit, and less social support tend to live longer. This result supported our hypothesis. Stable emotional responses [26], less acceptance of external support and smoking cessation [47, 48] predict individuals’ longevity to a certain extent. What concerned us was that neuroticism and psychoticism significantly predicted familial longevity. Lower neuroticism scores indicate more stability and emotional control and better mental health [27], while higher psychoticism tend to lead to longer lives for the family, this may be related to Chinese culture of respect the old and family pension model, the elderly represent authority in traditional Chinese families, they tend to be lonely, persistent and even stubborn.

Our results have implications for the promotion of healthy ageing. The Blue Book on Health for the Elderly: Research and Policy on Healthy Aging in China (2020) pointed out that, healthy aging is not only reflected in the length of life, but more importantly, the improvement of the quality of life. Therefore, the first is to update the understanding of the health of longevous elderly. Physical health and mental health are equally important, changing bad living habits, improving emotional management ability and promoting interpersonal support could help to comprehensively improve the health, well-being and quality of life of the elderly. Secondly, it is recommended to improve the social support system for the longevous elderly from the government and the community, build an equal and harmonious family intergenerational culture [66]. Furthermore, optimization of pension service model. We could gradually change from family pension model to community pension model or institution pension model, improve the level of health guidance, build a network of elderly health service facilities, promote the transformation from disease-based treatment model to the elderly-centered comprehensive care model, and to further improve the quality of elderly care services.

Meanwhile, our study suffered from limitations also. First, we selected the elderly who were in family pension model as the research subjects. However, due to the urbanization process in China, the pension models become various, and the number of elderly who are in community and institution pension model has gradually increased. Further study should expand the research sample from various pension models, and increase the sample size to collect comprehensive data from both individuals and their families. Then, limited to research energy, this study only focuses on the impact of personality traits, social support and other psychological factors on longevity. During the research process and result analysis, we realized that other factors, such as kindness, life attitudes and values, may also have effects on longevity. Future research can further expand the research content to obtain a more explanatory model.

## 5. Conclusions

This study revealed a predicted pattern in the interaction between psychosocial factors and longevity from both individual and family perspectives. We conclude that good emotional management, benign interpersonal support, and moderation of habits are important factors for individual longevity, and the intergenerational influence of personality is closely related to family longevity.

## Supporting information

S1 File Data underlying the findings. (DOCX)
