# Supplementary material for: Research on the prediction of longevity from both individual and family perspectives
Source: PLoS One. 2022 Feb 18;17(2):e0263992. doi: 10.1371/journal.pone.0263992 (PMC8856538; doi:10.1371/journal.pone.0263992)
Supplement: S1 File — (DOCX) [file pone.0263992.s001.docx]

**1 Demographics for Elderly Subsamples**

Frequency and Percentage of Demographic Variable

| Demographic Variable | | Longevous Elderly  (*n* = 62) | | Ordinary Elderly  (*n* = 57) | |
| --- | --- | --- | --- | --- | --- |
|  |  | Frequency | Percentage | Frequency | Percentage |
| Gender | Male | 14 | 22.6 | 30 | 52.6 |
|  | Female | 48 | 77.4 | 27 | 47.4 |
| Education | High school and beyond | 2 | 3.4 | 15 | 25.9 |
|  | Basic education | 16 | 25.4 | 34 | 60.3 |
|  | No schooling | 44 | 71.2 | 8 | 13.8 |
| Marital Status | Married | 12 | 18.6 | 39 | 69.1 |
|  | Widowed | 50 | 81.4 | 17 | 29.1 |
|  | Divorced | 0 | 0 | 0 | 0 |
|  | Single and others | 0 | 0 | 1 | 1.8 |
| Health Status | Good | 51 | 82.0 | 43 | 75.5 |
|  | Generally good | 9 | 14.8 | 13 | 22.6 |
|  | Poor | 2 | 3.3 | 1 | 1.9 |
| Habits | Smoking (yes) | 8 | 13.1 | 23 | 41.1 |
|  | Smoking (no) | 54 | 86.9 | 34 | 58.9 |
|  | Drinking (yes) | 18 | 29.5 | 28 | 49.1 |
|  | Drinking (no) | 44 | 70.5 | 28 | 50.9 |
| Household Income | ≤ RMB 2000 | 47 | 75.6 | 49 | 85.4 |
|  | ≥ RMB 2000 | 15 | 24.4 | 8 | 14.6 |

**2 Individual Differences on Psychosocial Variables**

2.1 Descriptive Statistics

| Variables | group | N | Mean | Std. Deviation | Std. Error Mean |
| --- | --- | --- | --- | --- | --- |
| Age | Longevous Elderly | 62 | 98.7419 | 5.24150 | .66567 |
|  | Ordinary Elderly | 57 | 73.4737 | 8.37536 | 1.10934 |
| Extraversion | Longevous Elderly | 61 | 53.0951 | 9.79582 | 1.25423 |
|  | Ordinary Elderly | 57 | 49.3965 | 9.19588 | 1.21802 |
| Neuroticism | Longevous Elderly | 61 | 40.9361 | 7.29036 | .93343 |
|  | Ordinary Elderly | 57 | 45.5509 | 9.56133 | 1.26643 |
| Psychoticism | Longevous Elderly | 61 | 49.7443 | 10.29175 | 1.31772 |
|  | Ordinary Elderly | 57 | 48.4579 | 10.93757 | 1.44872 |
| Objective Support | Longevous Elderly | 62 | 9.1935 | 4.46053 | .56649 |
|  | Ordinary Elderly | 57 | 10.2456 | 4.23118 | .56043 |
| Subjective Support | Longevous Elderly | 62 | 18.2258 | 8.30449 | 1.05467 |
|  | Ordinary Elderly | 57 | 21.7368 | 7.20745 | .95465 |
| Support Utilization | Longevous Elderly | 62 | 7.2258 | 3.25637 | .41356 |
|  | Ordinary Elderly | 57 | 8.0175 | 2.48202 | .32875 |
| Total Support | Longevous Elderly | 62 | 34.6452 | 13.76631 | 1.74832 |
|  | Ordinary Elderly | 57 | 40.0000 | 11.82008 | 1.56561 |

|  | | Levene’s Test for Equality of Variances | |  |  |  |  |  |  |
| --- | --- | --- | --- | --- | --- | --- | --- | --- | --- |
|  |  | F | Sig. | t | Sig. (2-tailed) | Mean Difference | Std. Error Difference | 95% Confidence Interval for Difference | |
|  |  |  |  |  |  |  |  | Lower Bound | Upper Bound |
| Age | Equal variances assumed | 11.288 | .001 | 19.896 | .000 | 25.26825 | 1.26999 | 22.75311 | 27.78340 |
|  | Equal variances not assumed |  |  | 19.531 | .000 | 25.26825 | 1.29374 | 22.69899 | 27.83752 |
| Extraversion | Equal variances assumed | .021 | .885 | 2.111 | .037 | 3.69859 | 1.75211 | .22832 | 7.16886 |
|  | Equal variances not assumed |  |  | 2.115 | .037 | 3.69859 | 1.74833 | .23580 | 7.16139 |
| Neuroticism | Equal variances assumed | 5.034 | .027 | -2.960 | .004 | -4.61481 | 1.55908 | -7.70277 | -1.52685 |
|  | Equal variances not assumed |  |  | -2.933 | .004 | -4.61481 | 1.57326 | -7.73444 | -1.49518 |
| Psychoticism | Equal variances assumed | .558 | .456 | .658 | .512 | 1.28637 | 1.95429 | -2.58436 | 5.15709 |
|  | Equal variances not assumed |  |  | .657 | .513 | 1.28637 | 1.95836 | -2.59309 | 5.16583 |
| Objective Support | Equal variances assumed | .188 | .665 | -1.317 | .190 | -1.05207 | .79865 | -2.63375 | .52962 |
|  | Equal variances not assumed |  |  | -1.320 | .189 | -1.05207 | .79687 | -2.63023 | .52610 |
| Subjective Support | Equal variances assumed | 1.694 | .196 | -2.453 | .016 | -3.51104 | 1.43107 | -6.34520 | -.67687 |
|  | Equal variances not assumed |  |  | -2.468 | .015 | -3.51104 | 1.42256 | -6.32844 | -.69363 |
| Support Utilization | Equal variances assumed | 5.009 | .027 | -1.482 | .141 | -.79174 | .53427 | -1.84984 | .26636 |
|  | Equal variances not assumed |  |  | -1.499 | .137 | -.79174 | .52831 | -1.83839 | .25491 |
| Total Support | Equal variances assumed | 1.189 | .278 | -2.267 | .025 | -5.35484 | 2.36195 | -10.03256 | -.67712 |
|  | Equal variances not assumed |  |  | -2.282 | .024 | -5.35484 | 2.34686 | -10.00290 | -.70678 |

**3 Demographics for Family Subsamples**

Frequency and Percentage of Demographic Variable

| Demographic Variable | | Longevous Families  *（n* = 186） | | Ordinary Elderly Families  *（n* = 246） | |
| --- | --- | --- | --- | --- | --- |
|  |  | Frequency | Percentage | Frequency | Percentage |
| Gender | Male | 108 | 58.1 | 136 | 55.1 |
|  | Female | 78 | 41.9 | 110 | 44.9 |
| Education | High school and beyond | 93 | 50.3 | 154 | 62.8 |
|  | Basic education | 81 | 43.3 | 83 | 33.8 |
|  | No schooling | 12 | 6.4 | 8 | 3.4 |
| Marital Status | Married | 151 | 81.0 | 212 | 86.1 |
|  | Widowed | 8 | 4.3 | 17 | 7.1 |
|  | Divorced | 0 | 0 | 3 | 1.3 |
|  | Single and others | 27 | 14.7 | 14 | 5.5 |
| Health Status | Good | 169 | 90.6 | 204 | 82.8 |
|  | Generally good | 15 | 8.3 | 38 | 15.5 |
|  | Poor | 2 | 1.1 | 4 | 1.7 |
| Habits | Smoking (yes) | 53 | 28.5 | 84 | 34.0 |
|  | Smoking (no) | 133 | 71.5 | 162 | 66 |
|  | Drinking (yes) | 65 | 34.9 | 103 | 41.7 |
|  | Drinking (no) | 121 | 65.1 | 143 | 58.3 |
| Household Income | ≤ RMB 2000 | 118 | 63.7 | 152 | 61.7 |
|  | ≥ RMB 2000 | 68 | 36.3 | 94 | 38.3 |

**4 Family Differences on Psychosocial Variables**

4.1 Descriptive Statistics

| Variables | group | N | Mean | Std. Deviation | Std. Error Mean |
| --- | --- | --- | --- | --- | --- |
|  |  |  |  |  |  |
| Age | Longevous Families | 186 | 50.4892 | 17.73536 | 1.30042 |
|  | Ordinary Elderly Families | 246 | 50.4634 | 15.33080 | .97746 |
| Extraversion | Longevous Families | 186 | 51.4919 | 9.76955 | .71827 |
|  | Ordinary Elderly Families | 246 | 51.8508 | 9.74913 | .62158 |
| Neuroticism | Longevous Families | 186 | 43.4432 | 9.20965 | .67711 |
|  | Ordinary Elderly Families | 246 | 46.1114 | 9.48013 | .60443 |
| Psychoticism | Longevous Families | 186 | 49.8157 | 10.38996 | .76389 |
|  | Ordinary Elderly Families | 246 | 48.4455 | 9.76968 | .62289 |
| Objective Support | Longevous Families | 186 | 10.7581 | 3.93880 | .28881 |
|  | Ordinary Elderly Families | 246 | 10.9268 | 3.88649 | .24779 |
| Subjective Support | Longevous Families | 186 | 23.7634 | 6.80047 | .49863 |
|  | Ordinary Elderly Families | 246 | 23.8130 | 6.16686 | .39318 |
| Support Utilization | Longevous Families | 186 | 8.0860 | 2.33496 | .17121 |
|  | Ordinary Elderly Families | 246 | 7.9472 | 2.57802 | .16437 |
| Total Support | Longevous Families | 186 | 42.6075 | 9.63225 | .70627 |
|  | Ordinary Elderly Families | 246 | 42.6870 | 9.95067 | .63443 |

|  | | Levene’s Test for Equality of Variances | |  |  |  |  |  |  |
| --- | --- | --- | --- | --- | --- | --- | --- | --- | --- |
|  |  | F | Sig. | t | Sig. (2-tailed) | Mean Difference | Std. Error Difference | 95% Confidence Interval for Difference | |
|  |  |  |  |  |  |  |  | Lower Bound | Upper Bound |
| Age | Equal variances assumed | 11.350 | .001 | .016 | .987 | .02583 | 1.59437 | -3.15956 | 3.10789 |
|  | Equal variances not assumed |  |  | .016 | .987 | .02583 | 1.62681 | -3.22493 | 3.17326 |
| Extraversion | Equal variances assumed | .004 | .948 | -.378 | .706 | -.35892 | .94960 | -2.22537 | 1.50753 |
|  | Equal variances not assumed |  |  | -.378 | .706 | -.35892 | .94988 | -2.22636 | 1.50852 |
| Neuroticism | Equal variances assumed | .254 | .614 | -2.928 | .004 | -2.66814 | .91137 | -4.45945 | -.87682 |
|  | Equal variances not assumed |  |  | -2.940 | .003 | -2.66814 | .90764 | -4.45245 | -.88383 |
| Psychoticism | Equal variances assumed | .708 | .401 | 1.402 | .162 | 1.37015 | .97710 | -.55034 | 3.29064 |
|  | Equal variances not assumed |  |  | 1.390 | .165 | 1.37015 | .98565 | -.56783 | 3.30812 |
| Objective Support | Equal variances assumed | .107 | .743 | -.444 | .657 | -.16876 | .37983 | -.91532 | .57780 |
|  | Equal variances not assumed |  |  | -.443 | .658 | -.16876 | .38054 | -.91690 | .57937 |
| Subjective Support | Equal variances assumed | .001 | .977 | -.079 | .937 | -.04957 | .62644 | -1.28084 | 1.18171 |
|  | Equal variances not assumed |  |  | -.078 | .938 | -.04957 | .63500 | -1.29817 | 1.19903 |
| Support Utilization | Equal variances assumed | .233 | .629 | .577 | .564 | .13887 | .24062 | -.33407 | .61181 |
|  | Equal variances not assumed |  |  | .585 | .559 | .13887 | .23734 | -.32766 | .60540 |
| Total Support | Equal variances assumed | .193 | .661 | -.083 | .934 | -.07946 | .95369 | -1.95393 | 1.79500 |
|  | Equal variances not assumed |  |  | -.084 | .933 | -.07946 | .94938 | -1.94579 | 1.78686 |

**5 Individual Prediction Model of Longevity**

5.1 Omnibus test of model coefficient

|  | Chi square | df | Sig. |
| --- | --- | --- | --- |
| Step | 6.310 | 1 | .012 |
| Block | 30.149 | 3 | .000 |
| Model | 30.149 | 3 | .000 |

| 5.2 Hosmer & Lemeshow test | | |  |
| --- | --- | --- | --- |
| Chi square | df | Sig. | |
| 11.949 | 8 | .153 | |

| 5.3 Classification table | | | | |  |
| --- | --- | --- | --- | --- | --- |
| Observations | | Estimate | | | |
|  |  | Groups | | Correct percentage | |
|  |  | Longevous Elderly | Ordinary Elderly |  |  |
| Groups | Longevous Elderly | 16 | 45 | 73.8 | |
|  | Ordinary Elderly | 39 | 17 | 69.6 | |
| Total percentage | |  |  | 71.8 | |

| 5.4 Variables in Model | | | | | | | | |
| --- | --- | --- | --- | --- | --- | --- | --- | --- |
|  | B | S.E. | Wald | df | Sig. | Exp(B) | 95% EXP(B) Confidence Interval | |
|  |  |  |  |  |  |  | Lower Bound | Upper Bound |
| Neuroticism | -.088 | .027 | 10.573 | 1 | .001 | .916 | .869 | .966 |
| Total Support | -.045 | .019 | 5.694 | 1 | .017 | .956 | .922 | .992 |
| Smoking | -1.800 | .507 | 12.590 | 1 | .000 | .165 | .061 | .447 |

**6 Family Prediction Model of Longevity**

6.1 Omnibus test of model coefficient

|  | Chi square | df | Sig. |
| --- | --- | --- | --- |
| Step | 4.242 | 1 | .039 |
| Block | 13.414 | 2 | .001 |
| Model | 13.414 | 2 | .001 |

| 6.2 Hosmer & Lemeshow test | | |  |
| --- | --- | --- | --- |
| Chi square | df | Sig. | |
| 11.447 | 8 | .178 | |

| 6.3 Classification table | | | | |  |
| --- | --- | --- | --- | --- | --- |
| Observations | | Estimate | | | |
|  |  | Groups | | Correct percentage | |
|  |  | Longevous Family | Ordinary Elderly Family |  |  |
| Groups | Longevous Family | 121 | 64 | 34.6 | |
|  | Ordinary Elderly Family | 194 | 45 | 81.2 | |
| Total percentage | |  |  | 60.8 | |

| 6.4 Variables in Model | | | | | | | | |
| --- | --- | --- | --- | --- | --- | --- | --- | --- |
|  | B | S.E. | Wald | df | Sig. | Exp(B) | 95% EXP(B) Confidence Interval | |
|  |  |  |  |  |  |  | Lower Bound | Upper Bound |
| Neuroticism | -.038 | .011 | 11.198 | 1 | .001 | .963 | .942 | .985 |
| Psychoticism | .021 | .010 | 4.209 | 1 | .040 | 1.021 | 1.001 | 1.042 |
